# Diagnosis and prognostic value of circDLGAP4 in acute ischemic stroke and its correlation with outcomes

**DOI:** 10.3389/fneur.2022.931435

**Published:** 2022-07-29

**Authors:** Ying Bai, Hui Ren, Yi Zhu, Xufeng Chen, Li Yang, Jiangyan Xia, Guofang Shu, Bing Han

**Affiliations:** ^1^Department of Pharmacology, School of Medicine, Southeast University, Nanjing, China; ^2^Department of Emergency, Jiangsu Province Hospital, Nanjing, China; ^3^Department of Anesthesiology, Zhongda Hospital, Southeast University, Nanjing, China; ^4^Department of Clinical Laboratory, Zhongda Hospital, Southeast University, Nanjing, China

**Keywords:** circDLGAP4, ischemic stroke, biomarker, diagnosis, prognosis

## Abstract

**Rationale and aims:**

Circular RNAs are a subclass of noncoding RNAs in mammalian cells and highly expressed in the central nervous system. Although their physiological functions are not yet completely defined, they are thought to promise as stroke biomarkers because of their stability in peripheral blood.

Sample Size Estimate: 222 participants.

**Methods and design:**

The plasma of patients with acute ischemic stroke (AIS) (*n* = 111) and non-stroke controls (*n* = 111) from November 2017 to February 2019 were enrolled in our research. The expression of circDLGAP4 in plasma was evaluated using real-time PCR.

**Study outcomes:**

In patients with AIS, circDLGAP4 was significantly decreased in comparison with non-stroke controls. The CircDLGAP4 level had a significant AUC of 0.7896 with 91.72% sensitivity and 64.83% specificity in diagnosing AIS. Furthermore, the circDLGAP4 level was related to smoking history and previous transient ischemic attack/stroke/myocardial infarction in all samples. The change rate in circDLGAP4 within the first 7 days showed an AUC curve of 0.960 in predicting an stroke outcome.

**Conclusion:**

circDLGAP4 could serve as biomarker for AIS diagnosis and prediction of stroke outcomes.

## Introduction

Stroke is the second highest cause of death globally and a leading cause of disability. Ischemic stroke caused by arterial occlusion is responsible for the majority of strokes (1–3). It is a leading cause of adult disability that can severely compromise the life quality of patients (4–6). Our previous study first demonstrated that circular RNA DLGAP4 has functional roles in the mouse stroke model and may serve as a novel therapeutic target for acute ischemic injury (7). With this background, the aim of our study was to identify and validate circDLGAP4 in patients with stoke and to investigate its potential as biomarkers for the diagnosis and prognosis of acute stroke (AIS).

## Method

### Standard protocol approval and patient consent

The ethics committee of the Affiliated Jiangsu Province Hospital approved this research protocol (approval ID: 2016-SR-235), and the participants or their legally authorized representative provided written informed consent to participate in the study.

### Study population

This was a retrospective case-control study. A total of 222 participants, included 111 non-stroke controls and 111 patients with AIS. Non-stroke controls were recruited from among those patients who underwent an annual medical examination at the hospital. Patients with AIS were recruited within 72 h of the symptom onset from the Jiangsu Province Hospital. The enrollment period was November 2017 to February 2019. Plasma of patients was collected immediately on admission before any treatment. All the patients had a final diagnosis of ischemic stroke as defined by an acute focal neurological deficit, in combination with a diffusion-weighted imaging-positive lesion on magnetic resonance imaging (MRI) or a new lesion on a delayed computed tomography scan. For all the non-stroke controls and the patients with AIS involved in the study, we excluded neurological, psychiatric diseases and malignant diseases, those who underwent surgery within the last 3 months, and those who took prior medication with low molecular weight or unfractionated heparin within the last month. Vitals were collected at hospital admission. After inclusion, clinical data were collected on standardized forms. The legally authorized representatives of the non-stroke controls and the patients with AIS provided written informed consent to participate in the study.

### Real-time PCR

The expression of circDLGAP4 was performed using an Applied Biosystems Real-Time PCR System. CircRNA was reversely transcribed using the HiScript Q RT SuperMix for the qPCR Kit (R123-01, Vazyme, Nanjing, China) and quantified *via* the AceQ qPCR SYBR Green Master Mix (Q141-02, Vazyme, Nanjing, China). The following primers were employed: circDLGAP4 forward primer: 5′-ACGGCTACTGGTTCCTAAAGC-3′; circDLGAP4 reverse primer: 5′-GGGGTCTTCTTATACGCCACT-3′.

## Result

### Patients with AIS had reduced circDLGAP4 levels

**Figure 1:**
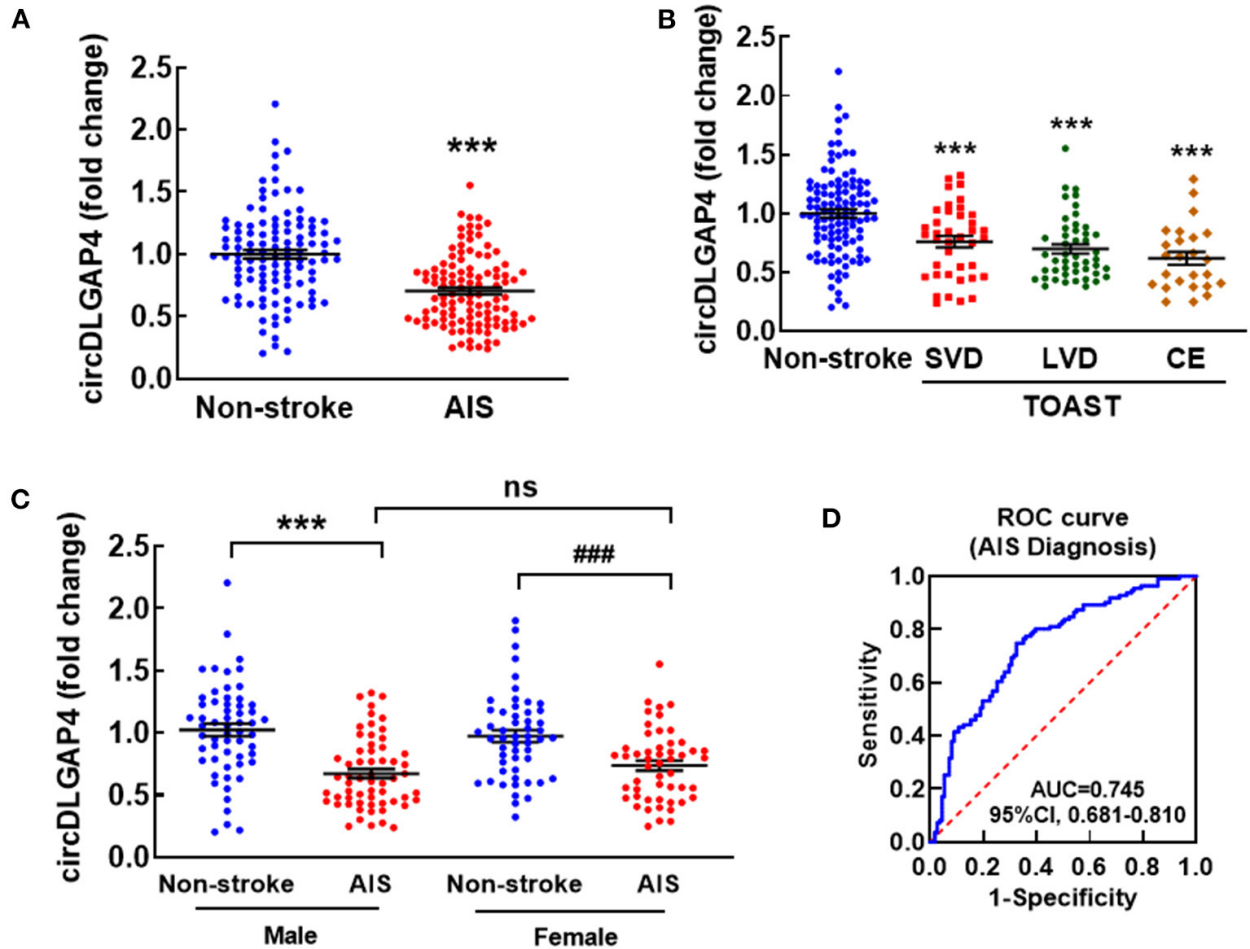
Downregulation of circDLGAP4 in patients with AIS. **(A)** circDLGAP4 expression levels in the plasma of patients with AIS increased compared with that in non-stroke controls. *N* = 111 individuals/group. (***p < 0.001, AIS vs. control using the Mann–Whitney test). **(B)** Relative plasma circDLGAP4 levels in patients with stroke categorized by TOAST criteria and non-stroke controls. The SVD group, *n* = 39; the LVD group, *n* = 47; the CE group, *n* = 25. ***p < 0.001 vs. non-stroke controls (1-way ANOVA followed by the Holm-Sidak *post-hoc* multiple comparisons test). **(C)** circDLGAP4 expression levels in the plasma of male and female patients with AIS decreased compared with that in male and female non-stroke controls. (***p < 0.001, male AIS vs. male control, ^###^*p* < 0.001, female AIS vs. female control using the Mann–Whitney Test). **(D)** ROC curves for circDLGAP4 to separate patients with AIS from non-stroke controls. AUC, area under curve; CE, cardio embolism; LVD, large vessel disease; ROC, receiver operating characteristic curve; SVD, small vessel disease; and TOAST, Trial of Org 10172 in Acute Stroke Treatment.

In patients with AIS, circDLGAP4 was significantly decreased compared with healthy controls (Figure 1A). The sociodemographic and clinical characteristics of replication samples are listed in Table 1. Each of the patients with AIS was assessed according to the TOAST (Trial of Org 10172 in Acute Stroke Treatment) criteria as being small vessel disease (*n* = 39), large vessel disease (*n* = 47), or cardio embolism (*n* = 25); there were decreased circDLGAP4 levels in all 3 of the subtype groups in comparison with the non-stroke control group (Figure 1B). CircDLGAP4 levels were significantly decreased in the plasma of male and female patients with AIS (Figure 1C). The analysis of the receiver operating characteristic (ROC) curve revealed that circDLGAP4 level had a significant area under the curve (AUC) of the 0.745 with 67.6% sensitivity and 74.8% specificity in diagnosing AIS (Figure 1D).

**Table 1:** Demographic and clinical characteristics of stroke and non-stroke samples.

	**Non-stroke**	**AIS**	**Statistical analysis (** ***P*** **)**
Demographic characteristics			
Total, n	111	111	1.000
Age, mean (SEM), years	68.06 (0.90)	70.95 (1.20)	0.055
Female, n (%)	51 (45.94)	51 (45.94)	1.000
Vascular risk factors, n (%)			
Hypertension	22 (19.82)	35 (31.53)	0.046
Smoking history	9 (8.11)	27 (24.32)	0.001
Hypercholesterolemia	25 (22.52)	35 (31.53)	0.723
Diabetes mellitus	53 (47.75)	84 (75.68)	<0.001
Previous TIA/stroke/MI	8 (7.21)	15 (13.51)	0.118
Laboratory parameters, mean (SEM)			
Glucose (mmol/L)	5.76 (0.15)	6.82 (0.22)	<0.001
AST (IU/L)	22.12 (0.72)	21.29 (0.61)	0.379
ALT (IU/L)	20.60 (1.03)	18.93 (0.85)	0.210
γ-GT (IU/L)	30.48 (2.64)	32.56 (2.64)	0.577
Total cholesterol (mmol/L)	4.71 (0.10)	4.54 (0.11)	0.251
Triglycerides (mmol/L)	1.55 (0.10)	1.44 (0.13)	0.511
HDL (mmol/L)	1.22 (0.03)	1.21 (0.03)	0.910
LDL (mmol/L)	2.80 (0.08)	2.70 (0.09)	0.389
WBC (10^9^/L)	6.49 (0.19)	7.48 (0.25)	0.002
RBC (10^12^/L)	4.31 (0.48)	4.52 (0.06)	0.006
Hb (g/L)	131.31 (1.52)	136.93 (1.76)	0.017
PLT (10^9^/L)	209.70 (7.22)	217.19 (9.02)	0.516
BUN (mmol/L)	5.69 (0.14)	5.86 (0.25)	0.564
Cr(μmol/L)	69.31 (1.60)	80.48 (3.10)	0.002
Total protein (g/L)	66.68 (0.51)	65.09 (0.79)	0.097
Albumin	39.94 (0.32)	39.01 (0.33)	0.046
Lp(a) (g/L)	282.05 (28.65)	276.76 (25.83)	0.892
INR	1.11 (0.04)	1.15 (0.03)	0.342
Prothrombin time(s)	11.93 (0.41)	12.09 (0.20)	0.734
APTT (s)	32.01 (0.39)	31.40 (0.36)	0.250
FIB (g/L)	3.73 (0.07)	3.98 (0.23)	0.306

*AIS, acute ischemic stroke; TIA, transient ischemic attack; MI, myocardial infarction; AST, aspartate transaminase; ALT, alanine transaminase; γ-GT, γ-glutamyl transpeptidase; HDL, high-density lipoprotein; LDL, low-density lipoprotein; WBC, white blood cell; RBC, red blood cell; Hb, hemoglobin; PLT, blood platelet; BUN, blood urea nitrogen; Cr, creatinine; Lp(a), lipoprotein (a); INR, International Normalized Ratio; APTT, activated partial thromboplastin time; FIB, fibrinogen*.

### Correlation between plasma circDLGAP4 levels and stroke severity

**Figure 2:**
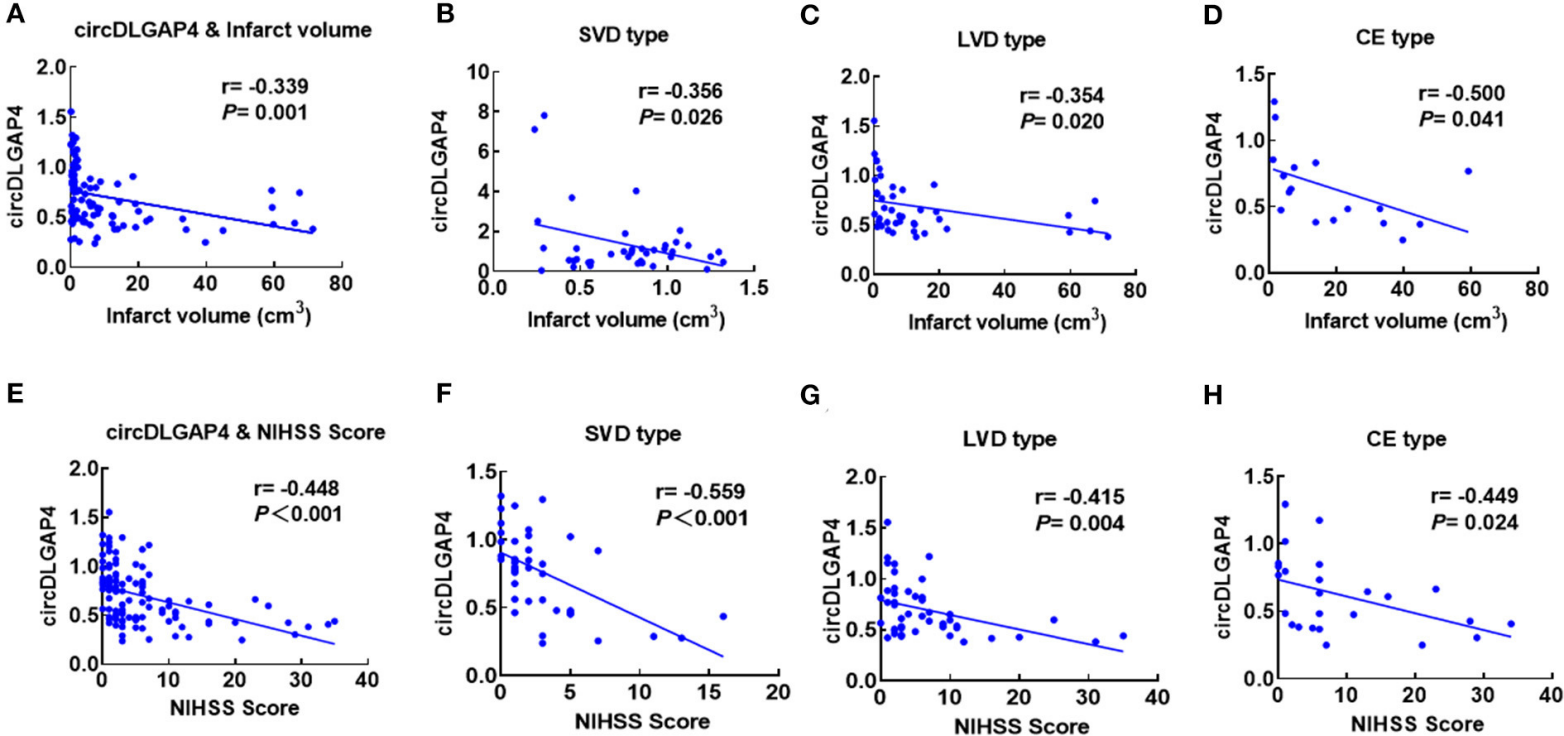
Correlation between plasma circDLGAP4 levels and stroke severity. **(A)** Correlation between plasma circDLGAP4 levels and infarct volume. Patients with AIS were recruited for infarct volume measurements. Correlation was estimated by calculating Pearson's correlation coefficient (*r* = −0.339, *p* = 0.001). **(B–D)** Correlations between circDLGAP4 levels in SVD **(B)**, LVD **(C)**, and CE **(D)** patients with stroke and infarct volume using Pearson's correlation coefficient (SVD: *r* = −0.356, *p* = 0.026; LVD: *r* = −0.354, *p* = 0.020; CE: *r* = −0.500, *p* = 0.041). **(E)** Correlation between plasma circDLGAP4 levels and the NIHSS score. Patients with AIS were recruited for infarct volume measurements. Correlation was estimated by calculating Pearson's correlation coefficient (r = −0.448, *p* < 0.001). **(F–H)** Correlations between circDLGAP4 levels in SVD **(F)**, LVD **(G)**, and CE **(H)** patients with stroke and NIHSS scores using Pearson's correlation coefficient (SVD: *r* = −0.559, *p <0.0*01; LVD: *r* = −0.415, *p* = 0.004; CE: *r* = −0.449, *p* = 0.024).

Furthermore, we observed a negative correlation between circDLGAP4 levels and the infarct volume (Pearson correlation coefficient *r* = −0.339, *p* = 0.001) (Figure 2A) and the NIHSS score (Pearson correlation coefficient *r* = −0.448, *p* < 0.001) (Figure 2E). Levels of circDLGAP4 are also significantly different based on patients' etiological classification as categorized by TOAST criteria in SVD, LVD, and CE stroke (Figures 2B–D). Negative correlations were observed between levels of circDLGAP4 and infarct volumes as well as NIHSS scores in SVD, LVD, and CE stroke (Figures 2F–H).

### CircDLGAP4 was an important factor in AIS diagnosis prediction

We further assessed the relationship between the circDLGAP4 level and the different vascular risk factors and found that the circDLGAP4 level was not related to major vascular risk factors, such as hypertension, diabetes, hypercholesterolemia, smoking history, and previous transient ischemic attack/smoke/myocardial infarction in all samples (Table 2). Next, we used the multivariable logistic regression models for AIS prediction. The AUC was calculated for two different models: Model 1 (comprising age and vascular risk factors) and Model 2 (comprising age, vascular risk factors, and the circDLGAP4 level). As shown in Table 3, the AUC of Model 2 was increased in comparison with the AUC Model 1 (Model 1: AUC = 0.733; Model 2: AUC = 0.819).

**Table 2:** The circDLGAP4 level in all samples with or without vascular risk factors.

	**circDLGAP4 level, mean (SEM)**			
	**With**	**Without**	**P**	**Statistical analysis**
Hypertension	0.872 (0.028)	0.791 (0.047)	0.141	*t-test*
Diabetes	0.884(0.035)	0.831 (0.032)	0.287	*t-test*
Hypercholesterolemia	0.846 (0.026)	0.895 (0.057)	0.516	*t-test*
Smoking history	0.860 (0.026)	0.807 (0.619)	0.412	*t-test*
Previous TIA/stroke/MI	0.839 (0.025)	0.949 (0.078)	0.156	*t-test*

*TIA, transient ischemic attack; MI, myocardial infarction*.

**Table 3:** The circDLGAP4 level in the multivariable logistic regression model for AIS diagnosis prediction.

**Variables**	**OR**	**95%CI**	***P***	**Prediction accuracy**
Model 1				
Age	1.017	0.989–1.045	0.231	0.733
Hypertension	0.642	0.330–1.248	0.191	
Diabetes	0.332	0.180–0.611	<0.001	
Smoking history	0.239	0.101–0.564	0.001	
Previous TIA/stroke/MI	0.706	0.256–1.880	0.486	
Model 2				
Age	1.012	0.983–1.043	0.422	0.819
Hypertension	0.726	0.345–1.529	0.399	
Diabetes	0.313	0.159–0.619	0.001	
Smoking history	0.209	0.082–0.537	0.001	
Previous TIA/stroke/MI	0.396	0.131–1.194	0.100	
circDLGAP4	0.042	0.014–0.127	<0.001	

*OR, odds ratio; CI, confidence interval; AUC, the area under the receiver operating characteristic curves; TIA, transient ischemic attack; MI, myocardial infarction*.

### Temporal expression profiles of circDLGAP4 according to modified Rankin Scale (mRS) at 3 months after stroke and their values in predicting an outcome

**Figure 3:**
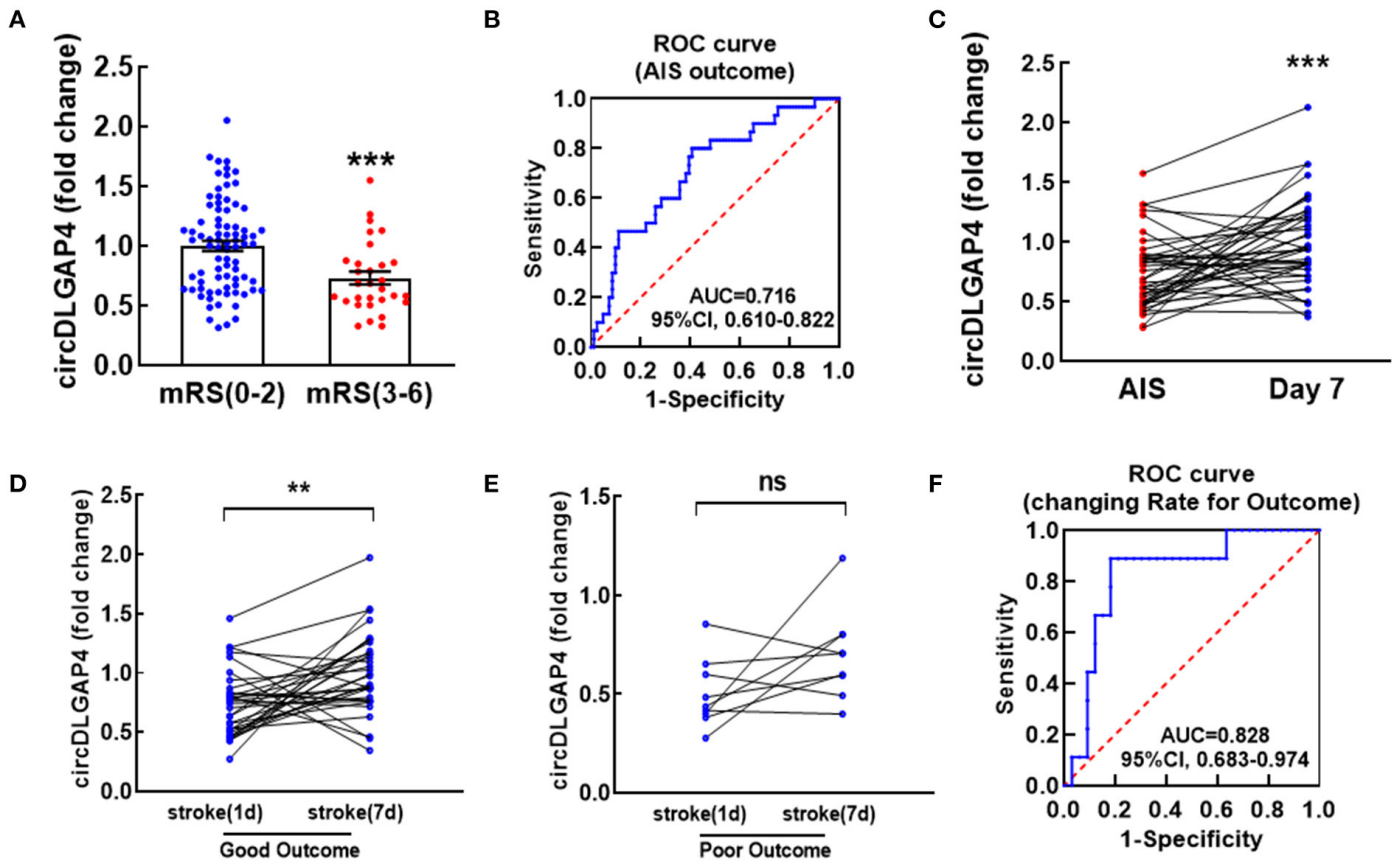
Temporal expression profiles of circDLGAP4 according to modified Rankin Scale (mRS) at 3 months after stroke and their values in predicting outcomes. **(A)** Relative plasma circDLGAP4 levels on the 1st day in patients with good (mRS 0-2) outcomes vs. poor (mRS 3-6) outcomes 3 months later; *n* = 81 in the good outcome group and *n* = 30 in the poor outcomes group. ***p < 0.0001 using the Mann–Whitney. **(B)** The ROC curve for individual circDLGAP4 on the 1^st^ day to separate good (mRS, 0–2) outcomes from poor (mRS, 3–6) outcomes 3 months later. **(C–E)** Relative plasma circDLGAP4 levels of total **(C)**, good outcome **(D)** and poor outcome **(E)** patients with AIS at Day 1 vs. Day 7 after treatment. ***p < 0.001 using the Wilcoxon matched-pairs signed rank test. AIS indicates acute ischemic stroke. **(F)** The ROC curve was evaluated by the changing rate (Δvalue/fold change on the 1st day) of patients with AIS. mRS: modified Rankin Scale.

Next, we further verified whether the baseline levels of circDLGAP4 were associated with the stroke outcome. A Mann–Whitney *U*-test revealed a significant difference in the baseline circDLGAP4 levels on the 1st day between the patients with AIS that eventually achieved good outcomes vs. poor outcomes (Figure 3A). Therefore, ROC curve analysis was performed to calculate the predictive power of the baseline circDLGAP4 level for the stroke outcome as the AUC was 0.716, with 59.3% sensitivity and 80.% specificity (Figure 3B). To further explore the changing level of circDLGAP4 during the acute phase of stroke up to the 7th day of hospitalization, plasma samples of the 42 patients with the stroke were collected on the 7th day after admission: the average circDLGAP4 level in plasma increased significantly by the 7th day (Figure 3C). There was significant increase in the patients with AIS with good outcomes (Figure 3D) but not in the patients with poor outcomes (Figure 3E). Furthermore, stroke outcomes were predicted by the changing rate of circDLGAP4 that further increased the predictive significance of circDLGAP4 with the AUC curve at 0.828 (Figure 3F).

### The significant difference between patients with good and poor outcomes with respect to age, hypertension, and previous TIA/stroke/MI

The demographic and clinical characteristics of patients with good and poor outcomes are listed in Table 4. We further detected the significant difference between patients with good and poor outcomes with respect to age, hypertension, diabetes, hypercholesterolemia, smoking, and previous transient ischemic attack/stroke/myocardial infarction. Moreover, by adding the circDLGAP4 level to the AIS outcome prediction Model 1 (including age and vascular risk factor), the AUC of the Model 2 (including age, vascular risk factors, and the circDLGAP4 level) was increased from 0.731 to 0.817 (Table 5).

**Table 4:** Demographic and clinical characteristics of good and poor outcome samples.

	**Good outcome**	**Poor outcome**	***P***
Total, n	81	30	/
Age, mean (SEM), years	68.42 (1.43)	77.80 (1.67)	<0.000
Female, n (%)	40 (51.25)	11 (36.67)	0.233
Vascular risk factors, n (%)
Hypertension	24 (29.63)	11 (36.67)	0.479
Smoking history	20 (24.69)	7 (23.33)	0.780
Hypercholesterolemia	25 (30.86)	10 (33.33)	0.804
Diabetes mellitus	61 (76.25)	23 (76.67)	0.989
Previous TIA/stroke/MI	9 (11.11)	6 (20.00)	0.308

*TIA, transient ischemic attack; MI, myocardial infarction*.

**Table 5:** The circDLGAP4 level in the multivariable logistic regression model for AIS outcome prediction.

**Variables**	**OR**	**95%CI**	***P***	**Prediction accuracy**
Model 1				
Age	0.921	0.877–0.967	0.001	AUC = 0.732
Hypertension	1.493	0.554–4.024	0.428	
Diabetes	0.695	0.224–2.160	0.529	
Hypercholesterolemia	1.382	0.516–3.701	0.520	
Smoking	1.676	0.549–5.122	0.365	
Previous TIA/stroke/MI	1.373	0.404–4.663	0.612	
Model 2				
Age	0.909	0.861–0.958	<0.000	AUC = 0.817
Hypertension	1.118	0.368–3.396	0.845	
Diabetes	0.674	0.194–2.345	0.535	
Hypercholesterolemia	1.297	0.440–3.822	0.637	
Smoking	1.493	0.456–4.887	0.508	
Previous TIA/stroke/MI	2.060	0.512–8.293	0.309	
circDLGAP4	18.850	3.205–110.853	0.001	

*OR, odds ratio; CI, confidence interval; AUC, the area under the receiver operating characteristic curves; TIA, transient ischemic attack; MI, myocardial infarction*.

## Discussion

Our previous study first demonstrated that circDLGAP4 was decreased in patients with AIS and had functional roles in the mouse stroke model (7). Furthermore, this study is the first to show that circDLGAP4 could serve as a potential biomarker for predicting stroke outcomes. To our knowledge, this is the first study to report that there was negative correlation between circDLGAP4 and infarct volume. Moreover, this is the first study to report that expression of circDLGAP4 was increased on 7th day of hospitalization and had the different change trends between good outcomes and poor outcomes. Our research indicates that patients with ischemic stroke with higher circDLGAP4 levels tend to have a lower risk of a poor functional outcome. Thus, circDLGAP4 may be a biomarker for predicting the functional outcome after stroke. In brief, our study has provided us with new insights into the function of circDLGAP4.

As we all know, the prediction of stroke has become increasingly important because of the serious consequences and the time pressure in the thrombolytic treatment of ischemic strokes. Identification of a specific biomarker for ischemic stroke can greatly contribute to prevention and treatment of patients (8). Noncoding RNAs, including short microRNAs, long ncRNAs, and circRNAs, are known as novel biomarkers that could be used as diagnostic (9, 10). CircRNAs are characterized by their covalently closed loop structures without a 5′ cap or a 3′ Poly A tail and have a special circular covalently bonded structure. Thus, circRNAs have higher tolerance to exonucleases. Because of their conservation and abundance, circRNAs may serve as a potential biomarker (11, 12). Our research found that there was a negative correlation between circDLGAP4 and infarct volume, as well as NIHSS Score. Accordingly, circDLGAP4 could serve as a specific biomarker for ischemic stroke and contribute to prevention and treatment of patients.

Moreover, identification of a biomarker for a stroke outcome can contribute to improved care of patients (8). Blood markers appeal as a predictor due to the ease of acquisition and relate to severity of stroke. Zhong et al. investigate a panel of protein markers in blood for their ability to predict stroke outcomes (13). In addition, higher omentin-1 levels at the baseline were negatively associated with a poor functional outcome among patients with ischemic stroke. Omentin-1 may represent a biomarker for predicting a poor functional outcome of patients with acute ischemic stroke (14). Furthermore, our previous study has demonstrated that the change rate of circFUNDC1, circPDS5B, and circCDC14A in plasma of patients with AIS could serve as a potential biomarker for predicting stroke outcomes (15). With this background, we further found that circDLGAP4 may be a new circRNA for predicting of stroke outcomes.

However, our study must be further confirmed in future studies. First, more ischemic patients in different areas should be involved to study for further verification. Secondly, the expression of circDLGAP4 in stroke subtype analysis of stroke outcomes should be studied in the future. Taken together, the findings of the current study have demonstrated for the first time that circDLGAP4 could serve as a potential biomarker for predicting stroke outcomes.

## Data availability statement

The raw data supporting the conclusions of this article will be made available by the authors, without undue reservation.

## Ethics statement

The studies involving human participants were reviewed and approved by the Ethics Committee of the Affiliated Jiangsu Province Hospital approved this research protocol (approval ID: 2016-SR-235). The patients/participants provided their written informed consent to participate in this study.

## Author contributions

YB, BH, GS, and JX contributed to the conception and design of the study, wrote sections of the manuscript, and performed the initial revision. YB, HR, YZ, and LY performed the literature review, wrote the first draft of the manuscript, and prepared the figure. All authors contributed to the final revision of the manuscript and figures and approved submitted version.

## Funding

This work was supported by grants from the National Natural Science Foundation of China (No. 81903591) and ZhiShan Scholar Program of Southeast University (No. 2242022R40059).

## Conflict of interest

The authors declare that the research was conducted in the absence of any commercial or financial relationships that could be construed as a potential conflict of interest.

## Publisher's note

All claims expressed in this article are solely those of the authors and do not necessarily represent those of their affiliated organizations, or those of the publisher, the editors and the reviewers. Any product that may be evaluated in this article, or claim that may be made by its manufacturer, is not guaranteed or endorsed by the publisher.

## References

[1] Campbell BCV De Silva DA Macleod MR Coutts SB Schwamm LH Davis SM. Ischaemic stroke. Nat Rev Dis Primers. (2019) 5:70. 10.1038/s41572-019-0118-8 31601801

[2] Sporns PB Hanning U Schwindt W Velasco A Minnerup J Zoubi T. Ischemic stroke: what does the histological composition tell us about the origin of the thrombus? Stroke. (2017) 48:2206–10. 10.1161/STROKEAHA.117.016590 28626055

[3] Winovich DT Longstreth WT Jr. Arnold AM Varadhan R Hazzouri AZA Cushman M. Factors associated with ischemic stroke survival and recovery in older adults. Stroke. (2017) 48:1818–26. 10.1161/STROKEAHA.117.016726 28526765 PMC5553701

[4] Creamer M Cloud G Kossmehl P Yochelson M Francisco GE Ward AB. Effect of intrathecal baclofen on pain and quality of life in poststroke spasticity. Stroke. (2018) 49:2129–37. 10.1161/STROKEAHA.118.022255 30354975 PMC6116794

[5] Liang Y Chen YK Deng M Mok VCT Wang DF Ungvar GS. Association of cerebral small vessel disease burden and health-related quality of life after acute ischemic stroke. Front Aging Neurosci. (2017) 9:372. 10.3389/fnagi.2017.00372 29180960 PMC5693845

[6] Joundi RA Cipriano LE Sposato LA Saposnik G Stroke Outcomes Research Working G. Ischemic stroke risk in patients with atrial fibrillation and CHA2DS2-VASc score of 1: systematic review and meta-analysis. Stroke. (2016) 47:1364–7. 10.1161/STROKEAHA.115.012609 27026630

[7] Bai Y Zhang Y Han B Yang L Chen X Huang R. Circular RNA DLGAP4 ameliorates ischemic stroke outcomes by targeting miR-143 to regulate endothelial-mesenchymal transition associated with blood-brain barrier integrity. J Neurosci. (2018) 38:32–50. 10.1523/JNEUROSCI.1348-17.2017 29114076 PMC6705810

[8] Mirzaei H Momeni F Saadatpour L Sahebkar A Goodarzi M Masoudifar A. MicroRNA: relevance to stroke diagnosis, prognosis, and therapy. J Cell Physiol. (2018) 233:856–65. 10.1002/jcp.25787 28067403

[9] Poller W Dimmeler S Heymans S Zeller T Haas J Karakas M. Non-coding RNAs in cardiovascular diseases: diagnostic and therapeutic perspectives. Eur Heart J. (2018) 39:2704–16. 10.1093/eurheartj/ehx165 28430919 PMC6454570

[10] Viereck J Thum T. Circulating noncoding RNAs as biomarkers of cardiovascular disease and injury. Circ Res. (2017) 120:381–99. 10.1161/CIRCRESAHA.116.308434 28104771

[11] Meng S Zhou H Feng Z Xu Z Tang Y Li P. CircRNA: functions and properties of a novel potential biomarker for cancer. Mol Cancer. (2017) 16:94. 10.1186/s12943-017-0663-2 28535767 PMC5440908

[12] Zhang HD Jiang LH Sun DW Hou JC Ji ZL. CircRNA: a novel type of biomarker for cancer. Breast Cancer. (2018) 25:1–7. 10.1007/s12282-017-0793-9 28721656

[13] Jickling GC Russo TL. Predicting stroke outcome: role of a biomarker panel. Neurology. (2019) 92:157–8. 10.1212/WNL.0000000000006715 30552297

[14] Xu T Zuo P Wang Y Gao Z Ke K. Serum omentin-1 is a novel biomarker for predicting the functional outcome of acute ischemic stroke patients. Clin Chem Lab Med. (2018) 56:350–5. 10.1515/cclm-2017-0282 28708570

[15] Zuo L Zhang L Zu J Wang Z Han B Chen B. Circulating circular RNAs as biomarkers for the diagnosis and prediction of outcomes in acute ischemic stroke. Stroke. (2020) 51:319–23. 10.1161/STROKEAHA.119.027348 31690252

